# Analysis of moral courage and related factors among undergraduate nursing students: a scoping review

**DOI:** 10.1590/0034-7167-2022-0225

**Published:** 2023-04-07

**Authors:** Romario Daniel Jantara, Jamila Geri Tomaschewski Barlem, Adrieli Jantara, Laurelize Pereira Rocha, Sabrina Santos da Rocha, Danubia Andressa da Silva Stigger

**Affiliations:** I Universidade Federal do Rio Grande. Rio Grande, Rio Grande do Sul, Brazil

**Keywords:** Nursing Students, Nursing Ethics, Courage, Morale, Review., Estudiantes de Enfermería, Ética en Enfermería, Coraje, Moral, Revisión., Estudantes de Enfermagem, Ética em Enfermagem, Coragem, Moral, Revisão.

## Abstract

**Objective:**

to analyze scientific evidence on moral courage and related factors among nursing undergraduate students.

**Method:**

the protocol of this scoping review was registered on Open Science Framework. A search was performed in five databases, according to the method provided by Joanna Briggs Institute Reviewers, the mnemonic strategy Population, Concept and Context, and a specific checklist.

**Results:**

a total of 2,812 results were identified, but only nine studies were eligible and presented three thematic approaches: Moral courage from the perspective of nursing students; Moral courage and related factors; and The teaching of moral courage in the training of nursing students. The factors related to moral courage include moral distress, moral sensitivity, age, and having a previous degree in the health field.

**Final Considerations:**

few studies were found with a low evidence level. Most were performed in developed countries, indicating some gaps that need to be addressed in the future.

## RESUMO

**Objetivo:**

analisar as evidências científicas sobre a coragem moral e fatores relacionados entre estudantes de graduação em enfermagem.

**Métodos:**

trata-se de uma scoping review com protocolo registrado no Open Science Framework, realizada em cinco bases de dados, conforme *método do* Joanna Briggs Institute Reviewers, estratégia mnemônica População, Conceito e Contexto, e checklist específico.

**Resultados:**

identificou-se 2.812 resultados, sendo que nove estudos foram elegidos. Três enfoques temáticos foram identificados: Percepção da coragem moral pelos estudantes de enfermagem; Coragem moral e fatores relacionados; e Ensino da coragem moral no âmbito da formação de estudantes de enfermagem. Dentre os fatores relacionados com a coragem moral, destacou-se o sofrimento moral, a sensibilidade moral, a idade e a presença de um diploma anterior na área da saúde.

**Considerações Finais:**

evidenciou-se poucas publicações e com baixo nível de evidência, sendo oriundas majoritariamente de países desenvolvidos, indicando que lacunas ainda precisam ser preenchidas.

## RESUMEN

**Objetivo:**

analizar las evidencias científicas sobre el coraje moral y factores relacionados entre estudiantes de graduación en enfermería.

**Métodos:**

se trata de un *scoping review* con protocolo registrado en el *Open Science Framework*, realizado en cinco bases de datos, conforme método de *Joanna Briggs Institute Reviewers*, estrategia mnemónica Población, Concepto y Contexto, y *checklist* específico.

**Resultados:**

se identificaron 2.812 resultados, siendo que nueve estudios fueron seleccionados. Tres enfoques temáticos fueron identificados: Percepción del coraje moral por los estudiantes de enfermería; Coraje moral y factores relacionados; y, la Enseñanza del coraje moral en el ámbito de la formación de estudiantes de enfermería. Entre los factores relacionados con el coraje moral, se destacaron el sufrimiento moral, la sensibilidad moral, la edad y la presencia de un título anterior en el área de la salud.

**Consideraciones Finales:**

se evidenciaron pocas publicaciones y con bajo nivel de evidencias provenientes, mayoritariamente, de países desarrollados, indicando que existen vacíos que necesitan ser llenados.

## INTRODUCTION

Health workers often experience situations that impose ethical challenges and may lead to professional burnout and moral distress^(1-2)^. Moral distress occurs when nurses cannot act according to their professional judgment and/or personal values due to external constraints or internal characteristics and may negatively impact them personally and professionally, affecting their physical, emotional, or psychological health. Ultimately, it may impact the quality of care delivery and even harm patients ^(3)^.

Nursing students may often experience violations of patient safety and dignity during clinical practice, and most are unable to act against these inappropriate practices. Even when some students manage to challenge these practices indirectly, patients may still find themselves at risk, and students continue to experience moral distress^(4)^.

Therefore, moral courage is a valuable virtue, responsible for helping nursing students and professionals in the process of making proper and ethical moral decisions, despite adverse consequences for themselves^(5)^. One of the requirements to establish a moral practice in nursing is the ability of nursing professionals to think and act morally and ethically^(6)^. Moral courage in the nursing field has many dimensions and levels, being expressed by nursing professionals through some attributes such as true presence, moral integrity, responsibility, honesty, advocacy, commitment, perseverance and personal sacrifice^(6)^.

In this context, one of the ways in which nurses can ensure patient safety is through actions imbued with moral courage. Although the acts that involve this virtue are not always noticed, they permeate everyday practice. Even though nursing students are taught to recognize their morals and values, they are not always able or choose to be brave and speak openly about inappropriate practices^(7)^.

However, numerous educational curricula in the health field simplify content, often excluding essential knowledge within ethics and morals. In addition, the current health scenario requires all health professionals to perform their duties from an ethical and moral perspective^(8)^. By grounding their actions on ethical values, health workers seem more protected when making decisions, benefiting patients and themselves. From this perspective, an exercise that can be effective in nursing practice is questioning, being able to recognize improper situations, and problematizing the context experienced in the routine of health services^(9)^.

A recent integrative literature review^(10)^ was the first attempt to synthesize knowledge concerning moral courage in nursing. Through a systematic search in databases, the review presented key concepts to understand moral courage, highlighting the definition and descriptions of moral courage, characteristics that a morally courageous nurse needs to present, and skills and actions. Another review^(4)^ addressed the issue with nursing students, though it was limited to exploring the factors that facilitate or inhibit the willingness of undergraduate nursing students to demonstrate moral courage in situations of poor patient care. The conclusion is that most students do not have the moral courage to intervene or speak out in these situations.

The Literature^(4,10)^ agrees on the need for new studies to adopt different methodologies to more deeply explore the phenomenon to understand better concepts and aspects of moral courage that remain unclear in the nursing field. The academic environment should also be addressed to identify how nursing students demonstrate moral courage and verify how these findings influence students’ attitudes, values, and behaviors^(4)^.

An extensive database search revealed no studies systematically analyzing scientific evidence on moral courage and related factors among nursing students. Hence, this study was developed considering that moral courage in the nursing field is an important moral virtue that can contribute to patient safety, teamwork, moral resilience, and alleviate moral distress^(2)^.

## OBJECTIVE

To analyze scientific evidence on moral courage and related factors among undergraduate nursing students.

## METHODS

This scoping review followed the method proposed by the Joanna Briggs Institute Reviewers. Scoping reviews are intended to identify the range of evidence available in the literature and help examine emerging evidence when there is no clarity, possibly leading to more specific questions. Five steps were followed: 1) identification of the research question; 2) identification of relevant studies; 3) selection of studies; 4) data analysis; 5) grouping, synthesis, and presentation of data^(11)^. This study protocol was registered in the Open Science Framework (https://osf.io/tsf8v), and its preparation followed the PRISMA-ScR verification checklist for this type of review^(12)^.

The PCC strategy was used, where (P) represents “population”; (C) - “concept”; and (C) - “context”. In this case, (P) - nursing students; (C) - moral courage and related factors; and (C) - undergraduate nursing courses. The guiding question established was: what is the scientific evidence available on moral courage and its related factors among students attending nursing undergraduate programs?

The following inclusion criteria were established: original studies published and available in English, Portuguese, or Spanish, addressing moral courage among nursing students. No time timeframe was determined for selecting the studies. Exclusion criteria were: duplicate studies, editorials, experience reports, theoretical essays, reflection studies, books, and reviews.

Two independent researchers searched and selected the studies between November and December 2021. A third independent researcher resolved disagreements. The databases were: Web of Science (Clarivate), Medical Literature Analysis and Retrieval System Online via US National Library of Medicine (MEDLINE/Pubmed), Scopus (Elsevier); Cumulative Index to Nursing and Allied Health Literature (CINAHL), and Latin American and Caribbean Literature on Health Sciences Information (LILACS).

The search strategies included a combination of the Boolean operators “AND” and “OR” with the following terms: “moral courage”, “moral strength”, “moral integrity”, “nursing students”, and “nursing student”. Because there is no exact descriptor of moral courage registered in the Medical Subject Headings (MeSH), this term and related terms provided in the literature^(10)^ were used to obtain the desired results. The search strategies are described in Chart 1.

**Chart 1:** Database search strategies, 2021

Databases	Search strategies
Web of Science	(“moral courage”) AND (“nursing students”) (ALL (“moral courage”) OR ALL (“moral strength”) OR ALL (“moral integrity”) AND ALL (“nursing students” OR “nursing student”)
MEDLINE/Pubmed	(“moral courage”) AND (“nursing students”) (“moral courage”) OR (“moral strength”)) OR (“moral integrity”)) AND (“nursing students”) OR (“nursing student”) ((((“moral courage”) OR (“moral strength”)) OR (“moral integrity”))) AND (“nursing students” OR “nursing student”)
Scopus	(“moral courage”) AND (“nursing students”) (ALL (“moral courage”) OR ALL (“moral strength”) OR ALL (“moral integrity”) AND ALL (“nursing students” OR “nursing student”)
CINAHL	(“moral courage”) AND (“nursing students”) ((((“moral courage”) OR (“moral strength”)) OR (“moral integrity”))) AND (“nursing students” OR “nursing student”)
LILACS	(“moral courage”) AND (“nursing students”) (“moral courage” OR “moral strength” OR “moral integrity”) AND (“nursing students”) (“moral courage” OR “moral strength” OR “moral integrity”) AND (“nursing student”)

The studies were exported to the reference manager software, Mendley®, immediately after identification to verify whether there were duplicate studies and work with the references found. The studies were selected according to the inclusion and exclusion criteria previously presented.

The studies’ level of evidence was assessed. Systematic reviews or meta-analyses of relevant clinical trials were classified as level I; evidence from randomized clinical trials was classified as level II; level III included non-randomized clinical trials; well-designed cohort and case-control studies were classified as level IV; systematic reviews of descriptive and qualitative research at level V; evidence from descriptive or qualitative studies at level VI; and expert opinions or expert committees not based on scientific research were classified at level VII^(13)^.

The studies were analyzed by reading and assessing their respective full texts. An instrument was designed to include study reference, year, country, periodical, level of evidence, objective, methodology, results, and conclusions. The results and conclusions were grouped and described according to each study’s thematic approach.

## RESULTS

**Figure 1:**
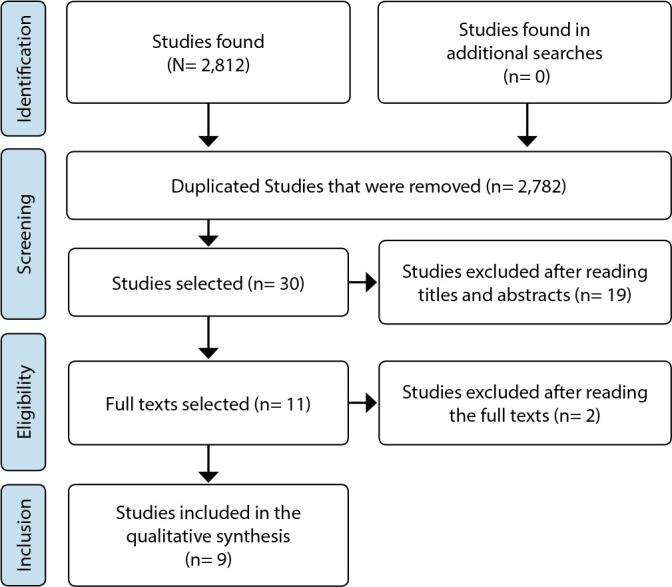
PRISMA-ScR flowchart for the selection of studies, 2021

The literature search resulted in 2,812 results. After removing duplicate studies, reading abstracts and titles, and full texts and applying inclusion and exclusion criteria, nine studies were included^(14-22)^ in this review. Figure 1 shows the selection process.

### Studies’ Characteristics

Regarding the year of publication, one study (11%) was published in 2014^(20)^, two (22%) in 2016^(14,22)^, two (22%) in 2018^(16,10)^, one (11%) in 2019^(21)^, one (11%) in 2020^(17)^, and two (22%) in 2021^(15,18)^. The origin and location of the studies were the United States of America^(17,21-22)^, Australia^(14)^, Iran^(19)^, the United Kingdom^(20)^, and the Philippines^(16)^. Additionally, two multicenter studies were identified, one carried out in Spain and Mexico^(15)^ and another in Spain, Finland, Iceland, Germany, Lithuania, and Ireland^(18)^.

Regarding the methodological design, six studies presented a cross-sectional quantitative approach^(15-19,21)^, and three presented a descriptive qualitative approach^(14,20,22)^. As for the level of evidence, all studies (100%) were classified with a level of evidence VI^(14-22)^. Regarding the publication language, the nine studies (100%) were written in English^(14-22)^. The synopsis of the studies containing an assigned code (ID), country of origin, journal, methodological design, participants, and evidence (EL) is shown in Chart 2.

**Chart 2:** Synopsis of studies addressing moral courage among undergraduate nursing students, 2021

ID	Country	Periodical	Methodological Design	Participants	EL
A1^(14)^	Australia	Nurse Education Today	Descriptive	Nursing students (n=9) and a recently graduated nurse (n=1)	VI
A2^(15)^	Spain and Mexico	International Journal of Environmental Research and Public Health	Cross-sectional	90 Spanish, 59 Mexican health workers; 56 medical and nursing students	VI
A3^(16)^	Philippines	International Journal Eating Disorders	Cross-sectional	293 Philippine undergraduate nursing students	VI
A4^(17)^	United States of America	The Journal of nursing education	Cross-sectional	Nursing students from three locations	VI
A5^(18)^	Spain, Philippines, Iceland, Germany, Lithuania, and Ireland	Nursing ethics	Cross-sectional	Graduate nursing students (n=1,796); nurse managers (n=538); and patients (n=1,327)	VI
A6^(19)^	Iran	Astra Salvensis	Cross-sectional	Nursing students (n= 185)	VI
A7^(20)^	United Kingdom	Nursing Ethics	Descriptive	Mentors/supervisors of nursing students (n=19)	VI
A8^(21)^	United States of America	SAGE open nursing	Cross-sectional	Nursing students (n=19) and nursing professors (n=8)	VI
A9^(22)^	United States of America	Nursing Ethics	Descriptive	Nursing students (n=15)	VI

Three main thematic approaches are found in the studies addressing moral courage in nursing students: Moral courage from the perspective of nursing students; Moral courage and related factors; and The teaching of moral courage in the training of nursing students. Table 1 presents the thematic approaches and the respective studies in which each approach was found.

**Table 1:** The main thematic approaches identified (n=9), Brazil, 2021

Thematic approach	Studies ID	n	%
Moral courage from the perspective of nursing students	A1	1	11.1
Moral courage and related factors	A2, A3, A4, A5, A6	5	55.6
The teaching of moral courage in the training of nursing students	A7, A8, A9	3	33.3

### Moral courage from the perspective of nursing students

The students^(14)^ understood moral courage to involve advocating for what they believed right when challenging an inadequate practice, regardless of difficulties or potential adverse consequences. However, participants added that moral courage comes from an instinctive reaction, such as intuitively knowing the right action. The students’ perception also emphasized a strong patient advocate identity. The students also considered the risk involved in demonstrating moral courage, as they could suffer negative consequences from their actions, characterized by ostracism and lateral violence. In addition, nurse supervisors and clinical facilitators should be highlighted as key individuals, as they may significantly impact the students’ decisions when challenging inappropriate practices. Supervisors and facilitators can provide support by reassuring and validating their actions, being physically present in the institutions, and offering practical advice on communicating concerns and solving problems^(14)^.

### Moral courage and related factors

Mexican professionals^(15)^ showed greater moral courage than Spanish professionals and students, evidencing cultural differences between countries. In addition, moral courage predicted anxiety disorder, acute stress disorder, and the presence of a mental disorder, considering that an individual may be susceptible to moral distress when s/he is unable to act in accordance with his/her moral values. In contrast, the purpose of life was considered to have a protective role in the emergence of psychopathologies. These results need to be further explored.

The frequency of moral distress was negatively related to moral courage, while the intensity of moral distress was positively correlated with moral courage. In addition, moral sensitivity (the ability to identify moral issues) was also positively related to moral courage in its different dimensions presented in the literature^(16)^.

Additionally, evidence^(17)^ shows that students reported mild levels of moral distress. Moral resilience was significantly correlated with moral courage, age, and students with a previous degree. Note^(18)^ that the mean obtained by the nursing students in moral courage self-assessment was 77.8 (scale from 0 to 100), with statistically significant differences between the six countries analyzed (i.e., Spain, Finland, Iceland, Germany, Lithuania, and Ireland).

Additionally^(18)^, the highest level of moral courage was associated with professional competence. An assessment of moral courage was performed among managers, patients, and students. Managers considered that nursing students presented a lower moral courage level than the students’ self-assessments. The students’ self-assessments were associated with being older, more experienced in the health field, having a previous degree in the health field, having a career plan in the nursing field, evaluating school performance as excellent, or being dissatisfied with the nursing profession. Moral courage was also associated with greater confidence in one’s ethical principles, considering nursing a profession appreciated in their country, and highly rated professional competence.

Further evidence^(19)^ showed a mean score of moral courage of 52.75 (between 15-75) among nursing students. The highest and lowest average dimensions were related to the moral factor and tolerance for threat. Educational level and having chosen nursing by interest were also positively associated with moral courage.

### The teaching of moral courage in the training of nursing students

One study carried out with mentor/supervisor nurses^(20)^ reported the experiences with nursing students failing the course, revealing a new perspective; moral courage from the mentoring perspective; addressing an understanding of failure; and what it means for mentors, how and why they fail students, and the culture of mentoring. Three key themes emerged: personal toll, sense of responsibility, and professional responsibility and being strong, elements associated with moral courage. The mentors who failed students felt that they had a sense of moral duty that extended to students and patients when deciding whether a student was apt to practice nursing; they felt morally obliged to fail a student inapt for the duty of providing care to people.

In an accelerated baccalaureate of Science in Nursing program (ABSN), the faculty observed significant growth in four of the five values related to moral courage among the respondents four weeks after the beginning and end of the program. Honesty, responsibility, justice, and compassion were all positively significant. The answers provided by 27 of the 29 ABSN students to the questionnaire at the end of the program ranged from respect and responsibility for 73%, compassion and honesty for 78%, and fairness for 82%. Teaching-learning activities to build moral courage values successfully promoted the program’s objectives^(21)^.

Nursing students needed moral courage to help homeless individuals regarding advance directives. For example, during a workshop intended to teach advance directives for the homeless, the students needed to show a higher level of moral courage before the workshop than at the time of the workshop^(22)^.

## DISCUSSION

Of the nine studies (100%) identified in this review, most (66.7%) were published in the last five years. It shows that although the focus of research on moral courage is recent, there is a growing trend. Such a trend may be related to recent moral distress and ethical conflicts experienced in the academic context and during the covid-19 pandemic, besides the discussions leading up to “Nursing Now”. Most of these studies (88.9%) were conducted in developed countries, showing a predominance within the scope of academic research, which may be explained by more frequent sources of public funding^(23)^.

The quantitative approach and cross-sectional design prevailed (66.7%), and all the studies (100%) were classified with evidence level VI(13), which indicates a weak level of strength^(24)^. Future studies are suggested to take advantage of more robust designs to provide evidence with a strong level of strength.

The studies presented three main approaches. One (11.1%) of the studies^(14)^ focused on the students’ perception concerning moral courage. The students considered moral courage to be an act of advocating for what is considered correct, emphasizing their role as patient advocates. A previous study^(4)^ corroborates such a finding, noting it is because nursing students are encouraged from the beginning of their studies to assume the patient advocate role. However, being a patient advocate requires courage, considering the risk of failure.

One study in this review^(14)^ shows that the students recognized the risk of adverse consequences when acting with moral courage. The daily routine of health services and intricate elements inherent to the organizational structure of these facilities and power relations challenge and discourage the development of moral and ethical actions according to the workers’ knowledge and moral standards, leading to moral distress^(25)^.

In this sense, the results of one of the studies included in the review indicate key elements and individuals that facilitate the process of acting with moral courage, i.e., supervisor and facilitator nurses^(14)^. Some elements facilitate the intervention process in nursing practice with a view to patient ethics and advocacy, and work environments are essential for this. Some facilitators concern with strengthened relationships established among health workers, the construction of a conducive working climate that facilitates the exercise of autonomy, the support provided by managers, an open and legitimate opportunity for dialogues, mastering clinical knowledge, continuous education, and the development of moral competences^(25)^.

Most studies (55.6%)^(15-19)^ focused on the prevalence and factors associated with moral courage, that is, on identifying the level of moral courage and its relationship with other objects of study in the sample composed of nursing students. Two studies (22.2%)^(18-19)^ reported the students’ level of self-assessment of moral courage, which was 77.8 (scale from 0 to 100) among students from six European countries^(18)^, and 52.75 among Iranian nursing students^(19)^.

Recently, an instrument was developed and validated to self-assess moral courage in nursing. It presents four dimensions: compassion and true presence, moral responsibility, moral integrity, and commitment to quality care. Nurses obtained high scores in this instrument measuring moral courage^(26)^, which aligns with the results presented in this review. However, the high scores nursing students self-assigned regarding moral courage may result from difficulties in assessing a concept that is very complex and abstract^(27)^.

The studies included in this review present statistical differences in moral courage among countries^(15,18)^ and the role of moral courage in predicting anxiety disorders, acute stress, and mental disorders^(15)^. Differences between countries can be explained by cultural differences and how ethics are taught^(18)^. No previous study has explored the predictive relationship between moral courage and anxiety and stress^(15)^. This relationship may be explained by moral distress, as professionals experience dissonance between expectations and what they can actually do in practice; those with greater moral courage but who are impeded from doing the right thing are more likely to develop a psychopathology^(28)^.

The analysis also showed a negative relationship between moral courage and moral distress and a positive relationship with moral sensitivity^(16)^. In addition, moral courage was positively associated with moral resilience^(17)^, level of professional competence, being older, being more experienced, having a degree in the health field, and characteristics related to the program and profession^(18)^, in addition to educational level and having chosen the nursing program with interest^(19)^.

The literature has already shown a negative and positive association between moral courage and moral distress, and moral resilience, respectively^(2)^. Likewise, the relationship between moral courage, moral sensitivity, and professional competence has already been established in previous studies, in addition to the relationship between moral courage and sociodemographic characteristics and those related to the program and profession^(29-31)^. However, a study selected in this review^(18)^ highlights the inconsistency of this result regarding satisfaction with the program, considering that moral courage was associated with assessing one’s school performance as excellent and being dissatisfied with the nursing profession. Thus, future studies should explore these factors more in-depth.

Regarding teaching moral courage in the context of training nursing students, three studies^(20-22)^ (33.3%) focused on this topic. The role of mentoring/supervising students to act with moral courage was emphasized^(20)^, besides the need for curricula to be based on moral courage values^(21)^ and the importance of working on specific themes within moral courage^(22)^.

Courage is essential for professionals in the health field. The multidisciplinary team includes nursing students who face situations that demand intentional attitudes of courage. Students show moral courage by choosing to overcome fears and fulfilling their duty in care delivery, advocating for patients, and standing up for themselves. In this context, educators should promote students’ courage and be supportive when they need the most, that is, in situations that demand courage^(7)^.

When students establish a relationship of mutual respect with their mentors, they are more confident to ask questions and act upon inappropriate practices. The opposite is also true. If the relationship is not based on mutual respect, students are more likely to remain silent and not challenge inappropriate practices. Similarly, when a student implements an intervention and receives positive feedback, s/he is more likely to repeat positive actions. In contrast, if a student is belittled, intimidated, or ignored, s/he may become omitted in the presence of malpractices^(4)^.

From this perspective, moral courage should be addressed before nurses start practicing the profession. One way to address moral courage is by using simulations involving moral dilemmas and other strategies to promote moral awareness and moral property^(8)^. In addition, the ability to make ethical decisions and act upon them must be encouraged^(32)^. Thus, nursing undergraduate programs should adopt pedagogical methodologies that value experiential learning, valuing previous literature that reinforces the concept of moral courage and its functioning in the academic milieu and clinical practice^(7)^.

Nursing students are the future of the profession, and thus, appreciating and discussing ethical problems during the training of these students provide them with tools to establish alternatives and implement necessary changes. Thus, nurses can cope with moral distress and nursing continues to be a critical and active profession, responsible for educational and health status, and respecting the lives of patients, students, and professionals^(33)^. Finally, work environments should welcome a critical reflection and value an open discussion, facilitating the practice of ethics^(32)^.

### Study Limitations

This review presents some limitations, including only papers written in Portuguese, English, or Spanish and excluding some types of publications. An additional limitation is that papers published in other sources were not included. These factors may have restricted access to other studies that might have provided relevant data.

### Contribuitions to Nursing

This study has important implications for nursing teaching and practice. The studies showed nursing students’ perceptions, their levels of moral courage, and the factors associated with this ethical virtue, besides teaching practices related to moral courage. These results support the education of nurses, enabling the discussion and implementation of practices aimed at promoting moral courage in the academic context and highlighting its importance in nursing education and practice.

## FINAL CONSIDERATIONS

This scoping review analyzes scientific evidence on moral courage among nursing undergraduate students. The results indicated a low level of evidence and strength. However, there is a growing trend in studies addressing this topic. All the studies originated in developed countries, except one, and reported a high level of moral courage. However, such a context may be different in developing countries where studies addressing this topic were not found, representing an important gap in the field.

Evidence on moral courage among nursing students has three thematic approaches that permeate aspects related to the students’ perception of moral courage, their level of moral courage and related factors, and the teaching of moral courage to students. Studies suggest that the factors related to moral courage include moral distress, moral sensitivity, age, and having a previous degree in the health field, among others. Furthermore, moral courage predicted anxiety disorder, acute stress disorder, and the presence of a mental disorder.

Although these studies are crucial for advancing knowledge in the field, important issues remain unclear, especially regarding the association between moral courage with mental disorders and satisfaction with the nursing program and profession. Hence, studies with more robust designs should be developed to clarify these aspects and explore moral courage among nursing students in more depth so that more accurate results are found with the level of evidence necessary to disseminate content on the topic and design more effective interventions.
